# Cutaneous adverse events to systemic antineoplastic therapies: a retrospective study in a public oncologic hospital^☆^

**DOI:** 10.1016/j.abd.2021.05.007

**Published:** 2021-11-26

**Authors:** William Queiroz Guimarães Wiegandt Ceglio, Marina Mattos Rebeis, Marcela Ferreira Santana, Denis Miyashiro, Jade Cury-Martins, José Antônio Sanches

**Affiliations:** a Department of Dermatology, Hospital das Clínicas, Faculty of Medicine, Universidade de São Paulo, São Paulo, SP, Brazil; b Instituto do Câncer do Estado de São Paulo, Hospital das Clínicas, Faculty of Medicine, Universidade de São Paulo, São Paulo, SP, Brazil

**Keywords:** Adverse events, Antineoplastics, Dermatology

## Abstract

**Background:**

Mucocutaneous adverse events are common during anticancer treatment, with variable consequences for the patient and their therapeutic regimen.

**Objective:**

To evaluate the most common adverse events, as well as the drugs associated with their appearance and the consequences for cancer treatment.

**Methods:**

A retrospective study was carried out through the analysis of patients treated at the Clinical Dermatology Unit of a public oncologic hospital.

**Results:**

A total of 138 patients with 200 adverse events were evaluated. The most commonly identified adverse events were nail and periungual changes (20%), papulopustular eruptions (13%), acneiform eruptions (12%), hand-foot syndrome (6.5%), hand-foot skin reaction (6%), and xerosis (6%). The most frequently associated antineoplastic treatment groups were classical chemotherapy (46.2%), target therapy (32.3%), and other non-antineoplastic drugs used in neoplasia protocols (16.5%). Of the total number of patients, 17.4% had their treatment suspended or changed due to a dermatological adverse event.

**Study limitations:**

Retrospective study and analysis of patients who were referred for specialized dermatological examination only, not allowing the assessment of the actual incidence of adverse events.

**Conclusion:**

A wide variety of dermatological manifestations are secondary to antineoplastic treatment with several different drugs resulting, not rarely, in the interruption or modification of therapeutic regimens.

## Introduction

Annually, 625,000 Brazilians are diagnosed with cancer, and approximately 230,000 die from it.^1^, ^2^ Fortunately, there have been several advances in the treatment of cancer patients, which include surgery, radiotherapy, and systemic therapies (classical chemotherapy, hormone therapy, target therapy, and immunotherapy), allowing a longer survival or even the cure of these patients.

It is noteworthy, however, that all these therapeutic modalities have potential adverse events (AEs). Systemic therapies that interfere with the growth of malignant also interfere with the proliferation of non-neoplastic cells, which may result in changes in the skin, its adnexa and mucous membranes.^3^

The most common adverse skin manifestations include anagen effluvium, xerosis, mucositis, hyperpigmentation, hand-foot syndrome, radiation recall, hypersensitivity reactions, extravasation injury, and ungual changes.^4^

Most adverse events are benign and self-limited, and do not require treatment interruption, although they often cause considerable physical and/or mental discomfort and impairment of the patient's quality of life.^5^, ^6^ Others result in severe complications, requiring dose reduction or even discontinuation of the involved drug.^6^, ^7^

However, not all skin changes developed throughout treatment are due to the direct action of the drugs. Other etiologies for these manifestations include infections, paraneoplastic syndromes, graft-*versus*-host disease, nutritional deficiencies, radiotherapy reactions, other malignancies, and cutaneous metastases.^6^

Thus, both dermatologists and oncologists need to be aware of these effects so they can be recognized and adequately managed. In many cases, preventive measures can be used before treatment starts, aiming to minimize the risk of these events.^7^

In the absence of a Brazilian series on the subject, this study aimed to report the most prevalent cutaneous manifestations secondary to systemic antineoplastic therapy in patients referred for dermatological evaluation in a public Brazilian oncologic hospital, as well as to describe the agents involved in their development and the impact of these AEs on cancer treatment.

## Methods

A retrospective observational study was carried out by reviewing the electronic medical records of patients seen at dermatological consultations and the Outpatient Dermatology Unit of the Instituto do Câncer do Estado de São Paulo Otávio Frias de Oliveira (ICESP) of the Hospital das Clínicas da Faculdade de Medicina da Universidade de São Paulo from January 1, 2014, to December 31, 2018.

All patients suspected of adverse mucocutaneous reactions related to systemic antineoplastic therapy were included. Those patients in which the hypothesis of skin rash secondary to antineoplastic systemic therapy was not confirmed or in which the etiological agent could not be identified by reviewing the medical record due to incomplete data or loss to follow-up were excluded.

Demographic characteristics (sex, age at the AE onset and type of neoplasm), systemic therapies (drug), and mucocutaneous AEs (type, duration and degree, according to the Common Terminology Criteria for Adverse Events v5.0)^8^ and the outcomes of cancer treatment (if there was suspension or modification due to the mucocutaneous AE) were extracted from the medical records.

Antineoplastic systemic therapies were divided into classical chemotherapy (the mechanism of which involves direct interference in some phase of the cell cycle, without cell specificity), target therapy (which acts through the binding of the drug to a protein or receptor only on target cells), immunotherapy (agents that act by increasing or changing the effectiveness of the individual's immune system), hormone therapy (a group of drugs that act on the hormone-induced growth of some cancers) and drugs that are complementary to the antineoplastic regimen (drugs that are part of treatment protocols but do not fit into the above categories). Subsequently, each antineoplastic treatment group was divided into subgroups, according to their classification. Classic chemotherapeutic agents were divided into antimetabolic agents, anthracyclines, alkylating agents, taxanes, alkaloids, antitumor antibiotics, topoisomerase I and II inhibitors, and unknown mechanisms; drugs used in target therapy in anti-EGFR, anti-VEGFR, anti-EGFR and anti-VEGFR, anti-PDGFR and c-Kit, a tyrosine kinase inhibitor, MEK inhibitor, and anti-HER-2; immunotherapy agents, in anti-PD-1; and hormone therapy, in aromatase inhibitor and androgen inhibitor.^9^

Non-parametric variables were described by the median, minimum and maximum values, and qualitative variables were described as absolute (N) and relative (%) frequencies. Data obtained through the research protocol were analyzed using STATA software, version 13 (STATA Corp., Texas, United States). This research was approved by the Núcleo de Pesquisa do ICESP and by the Ethics Committee of Faculdade de Medicina da Universidade de São Paulo (CEP/FMUSP), under registration number 1476/2019.

## Results

The medical records of 765 patients were reviewed, of which 138 (18.0%) met the inclusion criteria. The reasons for exclusion were: mucocutaneous manifestation unrelated to the use of antineoplastic drugs (n = 508; 81.0%), insufficient information in the medical record that made it impossible to establish the association between the treatment and the AE (n = 74; 11.8%), absence of lesion or presence of a residual lesion in the first dermatological consultation (n = 25; 4.0%), loss to follow-up due to death, absence or hospital discharge (n = 14; 2.2%) and lack of diagnostic definition due to a resolution of the clinical picture during follow-up and before its elucidation (n = 6; 1.0%). Of the included patients, 70 were female (50.7%) and 68 male (49.3%) with a median age of 54 years (ranging between 19 and 90 years). The malignant neoplasms that most frequently affected the patients were breast (n = 26; 18.8%), colon (n = 23; 16.7%) and thyroid (n = 10; 7.2%) neoplasms (Table 1).

**Table 1:** Demographic characteristics of patients.

Characteristic	n (%)
**Sex**	
Female	70 (50.7)
Male	68 (49.3)
**Age (in years)**	
Median (variation)	54 (19–90)
**Malignant Neoplasia (ICD-10)**	
Breast (C50)	26 (18.8)
Colon (C18)	23 (16.7)
Thyroid (C73)	10 (7.2)
Non-Hodgkin’s lymphoma (C83)	9 (6.5)
Lung (C34)	8 (5.8)
Hodgkin’s lymphoma (C81)	7 (5.0)
Myeloid leukemia (C92)	6 (4.3)
Testicular (C62)	5 (3.6)
Stomach (C16)	5 (3.6)
Rectal (C20)	5 (3.6)
Others^a^	33 (23.9)
**Total**	138 (100.0)

a Others: liver (3), multiple myeloma (3), skin (3), anus and anal canal (2), cervix (2), brain (2), esophagus (2), small intestine (2), ovary (2 ), soft tissue (2), prostate (2), kidney (2), lymphoid leukemia (2), bladder (1), body of uterus (1), oropharynx (1), pancreas (1), rectosigmoid (1).

As for the AEs, 67.4% of the patients had one AE (n = 93); in 22.5%, two AEs were identified concomitantly on dermatological evaluation (n = 31), in 8.0%, three AEs (n = 11) and in 2.1%, four AEs (n = 3). The median time between symptom onset and the first dermatological consultation was 25.5 days. Of the AEs, 27 were classified as grade I (19.6%), 16 as grade II (11.6%), 13 as grade III (9.4%), and 2 as grade IV (1.4%). Patients with grade IV AEs were not identified and there was no information in the medical file related to AE grade in 80 patients (58.0%).

Regarding systemic antineoplastic treatments, classical chemotherapy was the main drug group responsible for the AEs, comprising 73 cases (46.2%), followed by target therapy (n = 51; 32.3%), other non-antineoplastic drugs used together with chemotherapy protocols (n = 26; 16.5%), immunotherapy (n = 3; 1.9%) and hormone therapy (n = 2; 1.3%). In two cases (1.3%), it was not possible to identify the drug used because the patients were enrolled in a double-blind research protocol (Table 2).

**Table 2:** Classes and drugs used in systemic oncologic therapy, responsible for mucocutaneous adverse reactions.

	Drug	n	%	Total per class (%)
**Chemotherapy agent**				
Antimetabolites	Capecitabine	9	5.7	9.5
	Cytarabine	2	1.3	
	Gemcitabine	2	1.3	
	Fluorouracil	1	0.6	
	Methotrexate	1	0.6	
Anthracyclines	Doxorubicin	6	3.8	4.4
	Daunorubicin	1	0.6	
Alkylating agents	Cyclophosphamide	4	2.5	
	Cisplatin	3	1.9	
	Carboplatin	2	1.3	6.9
	Lomustine	1	0.6	
	Temozolomide	1	0.6	
Taxanes	Paclitaxel	20	12.7	15.2
	Docetaxel	4	2.5	
Alkaloids	Vincristine	3	1.9	1.9
Antitumor antibiotic	Bleomycin	8	5.0	5.0
Topoisomerase I inhibitor	Irinotecan	2	1.3	1.3
Topoisomerase II inhibitor	Etoposide	2	1.3	1.3
Unknown mechanism	Procarbazide	1	0.6	0.6
**Target therapy**				
Anti-EGFR	Panitumumab	22	13.9	18.9
	Cetuximab	3	1.9	
	Gefitinib	4	2.5	
	Erlotinib	1	0.6	
Anti-VEGFR	Axitinib	1	0.6	0.6
Anti-EGFR and Anti-VEGFR	Vandetanib	3	1.9	1.9
Anti-PDGFR and c-Kit	Imatinib	2	1.3	1.3
**Tyrosine kinase inhibitors**	Sorafenib	9	5.7	7.5
	Dasatinib	1	0.6	
	Pazopanib	1	0.6	
	Regorafenib	1	0.6	
**MEKi**	Trametinib	1	0.6	0.6
**Anti-HER-2**	Trastuzumab	2	1.3	1.3
**Immunotherapy**				
Anti-PD-1	Pembrolizumab	2	1.3	1.9
	Nivolumab	1	0.6	
**Hormone therapy**				
Aromatase inhibitor	Anastrozole	1	0.6	0.6
Androgen inhibitor	Apalutamide	1	0.6	0.6
Estrogen receptor modulator	Tamoxifen	1	0.6	0.6
**Complementary drugs**				
Corticosteroids	Dexamethasone	25	15.8	15.8
Granulocyte colony stimulator	Filgrastin	1	0.6	0.6
Not identified		2	1.3	1.3
Total		158	100	100

The most common group of AEs found in this series were nail and peri-ungual changes, affecting 40 patients and responsible for 20% of the dermatological toxicities in the study (Table 3). Among the nail and periungual changes, paronychia and onycholysis (15 and 13 patients, respectively) were the most frequently reported. Moreover, melanonychia (four patients), periungual pyogenic granuloma (three patients), onychodystrophy (two patients), onychomadesis (one patient), onychocryptosis (one patient), and subungual hematoma (one patient) were also reported (Table 4). The drugs most often implicated in these effects were epidermal growth factor receptor (EGFR) inhibitors, represented by panitumumab, gefitinib, and erlotinib, responsible for 50% of ungual changes (paronychia, periungual granulomas, onycholysis, onychodystrophy, and onychocryptosis). The second antineoplastic treatment group most often implicated in ungual and periungual changes were alkylating chemotherapy agents and taxanes, which caused ungual or periungual changes in 15 patients in this study: onycholysis (nine patients), melanonychia (two patients), onychomadesis (one patient), onychodystrophy (one patient) and subungual hematoma (one patient).

**Table 3:** Adverse mucocutaneous manifestations secondary to systemic oncologic therapies.

Mucocutaneous alteration	n (%)	Drugs involved
Ungual and periungual changes	40 (20)	Panitumumab (15), Paclitaxel (12), Docetaxel (3), Gefitinib (3), Doxorubicin (2), Daunorubicin (1), Erlotinib (2), Capecitabin (1), Doxorubicin and cyclophosphamide (1)
Papulopustular eruption	26 (13)	Panitumumab (15), Cetuximab (3), Sorafenib (2), Vandetanib (2), Gefitinib (1), Pazopanib (1), Trastuzumab (1), Dabrafenib and trametinib (1)
Acneiform eruption	24 (12)	Dexamethasone /prednisone (24)
Hand-foot syndrome	13 (6,5)	Capecitabine (5), Paclitaxel (3), Cisplatin (1), Cytarabine (1), Gemcitabine (1), Fluorouracil and oxaliplatin (1), Cisplatin and capecitabine (1)
Hand-foot reaction	12 (6)	Sorafenib (8), Axitinib (1), Cetuximab (1), Regorafenib (1), Panitumumab (1)
Xerosis	12 (6)	Panitumumab (6), Bleomycin (1), Erlotinib (1), Gefitinib (1), Pembrolizumab (1), Trastuzumabe (1), Not identified (1)^a^
Exanthemas	9 (4,5)	Carboplatin and paclitaxel (1), Dasatinib (1), Gemcitabine (1), Imatinib (1), Irinotecan and panitumumab (1), Panitumumab (1), Sorafenib (1), Not identified (2)^a^
Flagellate dermatitis	8 (4)	Bleomycin (8)
Trichomegaly	5 (2,5)	Panitumumab (4), Gefitinib (1)
Hypertrichosis	5 (2,5)	Panitumumab (4), Gefitinib (1)
Photosensitivity	4 (2)	Apalutamide (1), Capecitabine (1), Paclitaxel (1), Vandetanib (1)
Fissures	4 (2)	Panitumumab (4)
Anagen effluvium	3 (1,5)	Carboplatin and docetaxel (1), Docetaxel (1), Paclitaxel (1)
Pruritus	3 (1,5)	Paclitaxel (2), Pembrolizumabe (1)
Mucositis	3 (1,5)	Cytarabine and methotrexate (1), Sorafenib (1), Not identified (1)^a^
Acral lentigines	2 (1)	Capecitabine (1), Capecitabine, paclitaxel and gemcitabine (1)
Melasma aggravation	2 (1)	Paclitaxel (2)
Actinic keratosis irritation	2 (1)	Cisplatin (1), Vincristine and doxorubicin (1)
Acral hyperpigmentation	2 (1)	Capecitabine (1), Doxorubicin and cyclophosphamide (1)
Urticaria	2 (1)	Lomustine, vincristine and procarbazine (1), Temozolomide (1)
Others	17 (8,5)	Sorafenib (3), Imatinib (2), Paclitaxel (2), Doxorubicin, cyclophosphamide and paclitaxel (1), Tamoxifen (1), Etoposide and cisplatin (1), Vandetanib (1), Etoposide, vincristine, cyclophosphamide and doxorubicin (1), Nivolumab (1), Pembrolizumab (1), Filgrastim (1), Capecitabine (1), Pazopanib (1)

a Double-blind study.

**Table 4:** Ungual and periungual changes and the antineoplastic therapies involved.

Ungual or periungual changes	n (%^a^/%^b^)	Drugs involved
Paronychia	15 (37.5/7.5)	Panitumumab (11), Paclitaxel (1), Gefitinib (1), Erlotinib (1), Doxorubicin (1)
Onycholysis	13 (32.5/6.5)	Paclitaxel (7), Docetaxel (2), Capecitabine (1), Panitumumab (2), Doxorubicin (1)
Melanonychia	4 (10/2)	Paclitaxel (2), Daunorubicin (1), Doxorubicin and cyclophosphamide (1)
Periungual pyogenic granuloma	3 (7.5/1.5)	Panitumumab (2), Erlotinib (1)
Onychodystrophy	2 (5/1)	Docetaxel (1), Gefitinib (1)
Onychomadesis	1 (2.5/0.5)	Paclitaxel (1)
Onychocryptosis	1 (2.5/0.5)	Gefitinib (1)
Subungual hematoma	1 (2.5/0.5)	Paclitaxel (1)

a Percentage related to ungual changes.

b Percentage in relation to the other AEs.

The second most-often observed AE was a papulopustular eruption (PPE) (n = 26; 13.0%), mostly secondary to the use of EGFR inhibitors, due to their blocking by monoclonal antibodies (cetuximab and panitumumab), or due to the action of small molecules (erlotinib, gefitinib and vandetanib). PPE was also observed due to the action of non-selective multikinase inhibitors (sorafenib and pazopanib) and MEK inhibitors (trametinib) (Table 3).

Acneiform eruption (n = 24; 12.0%) was the third most frequently observed AE, being entirely secondary to the use of systemic corticosteroid therapy adjuvant to oncological therapies. Among acral eruptions, the Hand-Foot Syndrome (HFS) or erythrodysesthesia was present in 13 patients, accounting for 6.5% of the total AEs in this study. For these AEs, capecitabine was the most implicated drug (six patients), followed by paclitaxel (three patients). In turn, hand-foot reaction (HFR) was found in 12 patients in this study (6.0%), often associated with sorafenib (eight patients).

As for xerosis, it affected 12 patients (6.0%) and was mostly caused by EGFR inhibitors (panitumumab, erlotinib and gefitinib). Hair shaft changes were present in ten patients (four with trichomegaly, four with hypertrichosis, one with trichomegaly and hypertrichosis, and one with hair-shaft repigmentation) and accounted for 5.5% of the AEs. Altogether, they were caused by the EGFR inhibitors panitumumab (n = 7), gefitinib (n = 2), and vandetanib (n = 1). Exanthemas, present in nine patients and accounting for 4.5% of AEs, occurred during chemotherapy, targeted therapy and immunotherapy. Flagellate dermatitis occurred in eight patients (4.0%), all secondary to the use of bleomycin.

Other less frequently observed AEs included: photosensitivity (total of four patients), one case due to capecitabine, one due to paclitaxel, one case due to apalutamide and one case due to vandetanib; anagen effluvium (total of three patients) one due to carboplatin and docetaxel (one case), docetaxel (one case) and paclitaxel (one case); mucositis (total of three patients) caused by cytarabine and methotrexate (one case), sorafenib (one case) and unidentified (one case); acral lentigines (total of two patients) caused by capecitabine (one case) and capecitabine, paclitaxel and gemcitabine (one case); aggravation of melasma (total of two patients), both due to paclitaxel; actinic keratosis irritation (total of two patients), secondary to the use of cisplatin (one case) and vincristine and doxorubicin (one case); acral hyperpigmentation (total of two patients), due to capecitabine (one case) and doxorubicin and cyclophosphamide (one case); urticaria (total of two patients), triggered by lomustine, vincristine and procarbazide (one case) and by temozolomide (one case); androgenetic alopecia aggravated by the use of doxorubicin, cyclophosphamide and paclitaxel (one case); vesicant dermatitis caused by paclitaxel extravasation (one case); eczematous dermatitis secondary to tamoxifen (one case); erythema toxicum due to etoposide and cisplatin (one case); follicular pigmentation caused by vandetanib (one case); intertriginous eruption due to the use of etoposide, vincristine, cyclophosphamide and doxorubicin (one case); lipodystrophy caused by nivolumab (one case); lichen planopilaris secondary to imatinib use (one case); subacute lupus after paclitaxel use (one case); toxic epidermal necrolysis due to imatinib (one case); plaque psoriasis due to pembrolizumab (one case); keratosis pilaris due to sorafenib (one case); Sweet syndrome after filgrastin use (one case); perforating folliculitis due to sorafenib (one case); vitiligo repigmentation secondary to capecitabine use (one case); and finally, skin and hair depigmentation after the use of pazopanib (one case), individually comprising 0.5% of the sample.

Of the patients evaluated by the dermatology team, 24 (17.4%) had their antineoplastic treatment regimen changed or interrupted. Among them, nine had more than one concomitant AE (two EPP and paronychia; one PPE and xerosis; one PPE and HFR; one onycholysis and photosensitivity; one HFR and onycholysis; one xerosis and fissures; one PPE and periungual pyogenic granuloma; one HFS and vitiligo repigmentation). The AEs that most commonly resulted in this outcome were papulopustular eruption (seven cases), HFR (five cases), and HFS (five cases; Table 5). In six patients, the change or interruption occurred after specialized dermatological evaluation; of these, three cases were HFS (two caused by capecitabine and one due to the use of cytarabine), one due to papulopustular eruption (panitumumab), one case of photosensitivity (capecitabine) and the last case was secondary to pharmacoderma (tamoxifen). In 18 patients, treatments were interrupted or modified by the oncologist, prior to the dermatological consultation, one of which could be reintroduced after specialized evaluation (vandetanib, responsible for a papulopustular eruption).

**Table 5:** Drugs and their adverse reactions that culminated in drug suspension.

Adverse event	n^a^	Drug involved
Papulopustular eruption	7	Panitumumab (5), Vandetanib (1), Sorafenib (1)
Hand-foot reaction	5	Sorafenib (3), Panitumumab (1), Axitinib (1)
Hand-foot syndrome	5	Capecitabine (4), Cytarabine (1)
Paronychia	3	Panitumumab (3)
Xerosis	3	Panitumumab (3)
Photosensitivity	2	Capecitabine (1), Paclitaxel (1)
Flagellate dermatitis	1	Bleomycin (1)
Drug eruptions	1	Tamoxifen (1)
TEN	1	Imatinib (1)
Onycholysis	1	Paclitaxel (1)
Pyogenic granuloma	1	Panitumumab (1)
Fissures	1	Panitumumab (1)
Total	31	

a Total number of adverse events.

## Discussion

Although relevant for their incidence and morbidities, cutaneous toxicities are the object of few studies, usually in the context of phase II and III clinical trials.^5^ Epidemiological studies on the subject are scarce and use different populations and anticancer regimens, making it difficult to determine their incidence.^4^, ^5^

In this series, ungual and periungual changes were the most commonly observed AEs. In prospective studies of the overall incidence of AEs, their prevalence ranged from 2.9%–23.1%, being, in general, the third most frequent type of AE.^4^, ^5^ The facts that justify their position in this research include the lower frequency of referral of the most common adverse effects (anagen effluvium, for instance) and the fact that, in many cases, these reactions were not the reason for referral, but were accompanied by other AEs, thus being identified, registered in medical records and consequently included in this research.

In the specific literature on ungual and periungual changes secondary to antineoplastic treatments, taxanes, and target therapy are the drugs with the highest association.^10^ EGFR inhibitors are known to cause paronychia in 10%–15% of users.^11^ In extreme cases, periungual abscesses and pyogenic granuloma-like lesions may develop near the nail edge (the latter identified in three patients of this study, secondary to the use of panitumumab and erlotinib). Taxanes, classically associated with ungual changes, are responsible for up to 88% of these manifestations during their use, which can interfere with the daily activities of up to 43% of patients.^3^ The most often reported effects in the literature regarding this last class are onycholysis, Beau’s lines, nail hyperpigmentation, leukonychia, acute paronychia, and subungual and splinter hemorrhages.^3^, ^12^

Also regarding EGFR inhibitors, involved in 18.9% of the total of AEs in this general series, they were responsible for 73.1% of the papulopustular eruptions in the present study. It is the most common AE of this oncologic therapy, occurring in 50%–100% of patients.^11^, ^13^, ^14^

Acneiform eruption, a very frequent adverse reaction after the use of corticosteroids, was reported in 24 of the patients in this series, secondary to the use of dexamethasone and prednisone, adjunct to antineoplastic regimens. There are little data in the literature about this AE in this context, probably due to its use in almost all chemotherapy regimens and also because it is a very commonly expected AE.

The HFS, present in 13 patients, accounted for 6.5% of the total AEs in the study, classified as grade I (six patients), grade II (one patient), grade III (two patients) and not graded in four cases. This AE was responsible for the interruption of cancer treatment in two patients (grade I and grade II, respectively) and modification of the chemotherapy dose in three cases (two grade III cases and one case not graded), showing the impact on the patient's quality of life and the importance of recognizing this toxicity. Classically secondary to conventional chemotherapy agents (doxorubicin, capecitabine, cytarabine, 5-fluorouracil, and docetaxel), in this study, capecitabine was the most often implicated drug (six patients), followed by paclitaxel (three patients). The literature shows an incidence of HFS of 50%–60% in patients using capecitabine.^12^ HFR, despite sharing the same physiopathogenic hypothesis as HFS, is clinically quite distinct, representing 20%–40% of the AEs of multikinase inhibitors in the literature.^10^, ^15^, ^16^ In this series, it was one of the most frequently observed AEs (Table 4), as well as a frequent cause of interruption or modification of the chemotherapy regimen (Table 5).

With an incidence ranging from 1%–84%, xerosis is a common AE.^10^ Although it can develop during the use of classic chemotherapy, its occurrence has become more problematic with the use of target therapies, due to the greater severity of the condition and the chronicity of their use. One of the groups with the greatest association is the anti-EGFR, which can cause xerosis in up to 35% of the patients and, in this research, was responsible for two-thirds of the cases. Additionally, this AE can progress to xerotic eczema and painful fissures on fingers and toes (present in four patients in this study), with a great impact on the patients' quality of life.^11^ In some cases, it even requires treatment modification, as occurred in three patients in this study.

Exanthematous reactions can occur in all modalities of systemic antineoplastic treatment. However, data in the literature regarding exanthematous reactions are quite heterogeneous, which makes their interpretation difficult.^10^ Moreover, they are often triggered by non-drug-related causes. In the present study, exanthematous reactions were associated with chemotherapy and targeted therapy.

Flagellate dermatitis, when pharmacologically induced, is due to the use of bleomycin, with its occurrence ranging from 8%–66%.^17^ In agreement with the literature data, all cases of flagellate dermatitis in this research were due to the use of this chemotherapy agent.

Hair shaft changes characteristically occur with the use of anti-EGFR (having occurred exclusively in this study due to the use of the anti-EGFRs panitumumab, gefitinib, and vandetanib, thus corroborating current information). Literature data indicate that they are observed in up to 80% of patients after the sixth month of treatment. These include manifestations such as alopecia and changes in hair growth rate, thickness, shape, and, more rarely, hair shaft pigmentation.^18^

Photosensitivity is not a prevalent AE in epidemiological studies, a finding consistent with the low number of cases present in this study.^4^, ^5^ It can be caused by both classical chemotherapy and target therapy. In the first group, those with the highest association are dacarbazine, 5-fluorouracil, tegafur, and capecitabine; in the second, vandetanib, vemurafenib, and nivolumab.^6^, ^19^, ^20^ In the present study, representatives of both classes were found to be responsible for this adverse effect (with two cases secondary to classic chemotherapy, due to the use of capecitabine and paclitaxel and one case due to the use of target therapy with vandetanib). Additionally, the hormone therapy drug apalutamide was identified as the cause of photosensitivity in one patient. Despite the little occurrence in this study, this AE motivated the suspension of the antineoplastic regimen in two patients, totaling 8.3% of the interrupted therapy cases. It is noteworthy that several other drugs used in antineoplastic treatment can induce photosensitivity.^6^, ^19^

Common adverse events of traditional chemotherapy, such as anagen effluvium, which can affect approximately 75% of these patients, and mucositis, which affects 40%–70% of patients, were underrepresented in this study.^3^, ^4^, ^5^, ^6^ This finding may be explained by the oncologist's greater familiarity with these AEs and by their reversibility after the treatment, resulting in a lower rate of referral to the dermatologist.

The limitations of this study include its design, which prevented the calculation of the prevalence of adverse reactions, and the large number of patients excluded due to insufficient information in the medical records (n = 74; 11.8%). Because the database comprises only patients referred for dermatological evaluation, the number of less common adverse reactions may have been inflated because they are less known by oncologists. These limitations demonstrate the need to carry out prospective studies in users of systemic antineoplastic therapies aiming to obtain the actual prevalence of each adverse event due to the different therapeutic options employed.

Another limitation was the low rate of graded AEs, which has become a systematic practice in the service only more recently. This recording allows documenting and classifying the toxic effects of oncologic therapies, emphasizing that an accurate grading system is important both when starting the treatment of AEs and to assess the clinical response.^3^

The effect of AEs on the quality of life of patients was not considered in this study, but the importance of introducing this assessment as a routine is highlighted, aiming to obtain subsidies for more efficient and humanized care.

A low rate of AEs related to immunotherapy was observed in this sample, a phenomenon that can be explained by the non-availability of this therapeutic modality in the Brazilian Unified Health System (SUS, *Sistema Único de Saúde*) during the study period, with the included cases originating from research protocols.

Finally, one should emphasize the importance of establishing a common terminology among the several health professionals involved in patient management, allowing the standardization of the treatment and dose adjustments according to the severity of the clinical expression of the AEs. The National Cancer Institute Common Terminology Criteria for Adverse Events (NCI-CTCAE) is a standardized tool for the classification of the toxic effects of oncologic therapies.

## Conclusion

Mucocutaneous adverse events secondary to antineoplastic therapies commonly affect patients and often result in changes in oncologic treatment. With the development of new drugs, more adverse events have been identified. This fact makes it essential for dermatologists to be continually updated to achieve maximum diagnostic accuracy and the best therapeutic approach to AEs, with an ideal balance between oncological outcome and quality of life for the patient.

## Financial support

None declared.

## Authors' contributions

William Queiroz Guimarães Wiegandt Ceglio: Approval of the final version of the manuscript; design and planning of the study; drafting and editing of the manuscript; collection, analysis, and interpretation of data; critical review of the manuscript.

Marina Mattos Rebeis: Approval of the final version of the manuscript; design and planning of the study; drafting and editing of the manuscript; collection, analysis, and interpretation of data; critical review of the manuscript.

Marcela Ferreira Santana: Approval of the final version of the manuscript; design and planning of the study; drafting and editing of the manuscript; collection, analysis, and interpretation of data; critical review of the manuscript.

Denis Miyashiro: Approval of the final version of the manuscript; design and planning of the study; critical review of the manuscript.

Jade Cury-Martins: Approval of the final version of the manuscript; design and planning of the study; critical review of the manuscript.

José Antônio Sanches: Approval of the final version of the manuscript; design and planning of the study; critical review of the manuscript.

## Conflicts of interest

None declared.
